# *catena*-Poly[[[aqua­bis(1*H*-imidazole-κ*N* ^3^)copper(II)]-μ-naphthalene-1,4-dicarboxyl­ato-κ^2^ *O* ^1^:*O* ^4^] dihydrate]

**DOI:** 10.1107/S1600536808018515

**Published:** 2008-06-21

**Authors:** Jun-Hua Li, Jing-Jing Nie, Duan-Jun Xu

**Affiliations:** a Department of Chemistry, Zhejiang University, People’s Republic of China

## Abstract

In the title compound, {[Cu(C_12_H_6_O_4_)(C_3_H_4_N_2_)_2_(H_2_O)]·2H_2_O}_*n*_, the Cu^II^ cation is coordinated by two naphthalene-1,4-dicarboxyl­ate (naph) dianions, two imidazole mol­ecules and one water mol­ecule in a distorted square-pyramidal geometry. The Cu—O bond distance in the apical direction is 0.509 (3) Å longer than the mean Cu—O bond distance in the basal plane. The naph dianion bridges two Cu^II^ cations, forming a one-dimensional polymeric chain. The coordinated water mol­ecule is hydrogen-bonded to the carboxylate groups and imidazole ligands of adjacent polymeric chains, forming a three-dimensional supra­molecular structure. No π–π stacking is observed in the crystal structure. One solvent water molecule is disordered equally over two positions.

## Related literature

For general background, see: Su & Xu (2004 ▶); Li *et al.* (2005 ▶). For related structures, see: Derissen *et al.* (1979 ▶); Li *et al.* (2008 ▶).
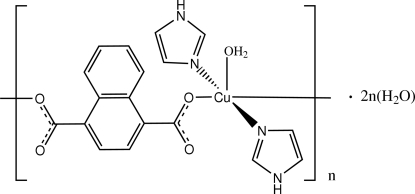


## Experimental

### 

#### Crystal data

[Cu(C_12_H_6_O_4_)(C_3_H_4_N_2_)_2_(H_2_O)]·2H_2_O
*M* *_r_* = 467.92
Monoclinic, 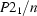

*a* = 12.571 (2) Å
*b* = 14.698 (3) Å
*c* = 12.636 (2) Å
β = 119.011 (6)°
*V* = 2041.8 (6) Å^3^
*Z* = 4
Mo *K*α radiation
μ = 1.12 mm^−1^
*T* = 295 (2) K
0.33 × 0.30 × 0.24 mm

#### Data collection

Rigaku R-AXIS RAPID IP diffractometer
Absorption correction: multi-scan (*ABSCOR*; Higashi, 1995 ▶) *T*_min_ = 0.660, *T*_max_ = 0.765
23205 measured reflections
3989 independent reflections
3251 reflections with *I* > 2σ(*I*)
*R*_int_ = 0.043

#### Refinement

*R*[*F* ^2^ > 2σ(*F* ^2^)] = 0.036
*wR*(*F* ^2^) = 0.097
*S* = 1.06
3989 reflections
280 parameters
H-atom parameters constrained
Δρ_max_ = 0.50 e Å^−3^
Δρ_min_ = −0.39 e Å^−3^

### 

Data collection: *PROCESS-AUTO* (Rigaku, 1998 ▶); cell refinement: *PROCESS-AUTO*; data reduction: *CrystalStructure* (Rigaku/MSC, 2002 ▶); program(s) used to solve structure: *SIR92* (Altomare *et al.*, 1993 ▶); program(s) used to refine structure: *SHELXL97* (Sheldrick, 2008 ▶); molecular graphics: *ORTEP-3 for Windows* (Farrugia, 1997 ▶); software used to prepare material for publication: *WinGX* (Farrugia, 1999 ▶).

## Supplementary Material

Crystal structure: contains datablocks I, global. DOI: 10.1107/S1600536808018515/sg2252sup1.cif

Structure factors: contains datablocks I. DOI: 10.1107/S1600536808018515/sg2252Isup2.hkl

Additional supplementary materials:  crystallographic information; 3D view; checkCIF report

## Figures and Tables

**Table 1:** Selected bond lengths (Å)

Cu—N1	1.992 (2)
Cu—N3	1.990 (2)
Cu—O1	1.9819 (17)
Cu—O3^i^	2.0116 (17)
Cu—O5	2.506 (2)

Symmetry code: (i) 
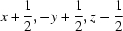
.

**Table 2:** Hydrogen-bond geometry (Å, °)

*D*—H⋯*A*	*D*—H	H⋯*A*	*D*⋯*A*	*D*—H⋯*A*
O1*W*—H1*A*⋯O3	0.87	1.99	2.846 (3)	170
O1*W*—H1*B*⋯O4^ii^	0.89	1.93	2.789 (3)	162
O2*WA*—H2*A*⋯O1*W*	0.91	1.97	2.828 (13)	155
O2*WB*—H2*C*⋯O2*WA*	0.85	1.55	2.156 (16)	126
N2—H2*N*⋯O1*W*^iii^	0.86	1.96	2.798 (4)	165
N4—H4*N*⋯O5^iv^	0.86	2.02	2.866 (3)	166
O5—H5*A*⋯O2^ii^	0.85	1.90	2.716 (3)	162
O5—H5*B*⋯O4^v^	0.85	1.95	2.791 (3)	172
C17—H17⋯O2^vi^	0.93	2.50	3.389 (4)	160

Symmetry codes: (ii) 
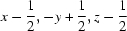
; (iii) 
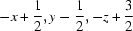
; (iv) 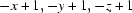; (v) 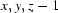; (vi) 
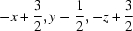
.

**Fig. 1.**
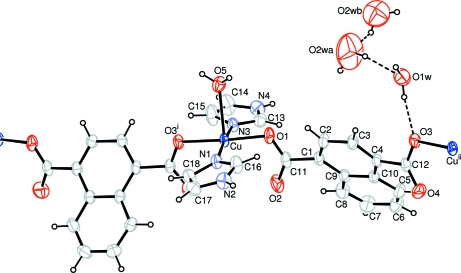
A segment of the polymeric chain of the title compound with 30% probability displacement ellipsoids (arbitrary spheres for H atoms); dashed lines indicate hydrogen bonding [symmetry codes: (i) x + 1/2, -y + 1/2, z - 1/2; (ii) x - 1/2, -y + 1/2, z + 1/2].

**Fig. 2.**
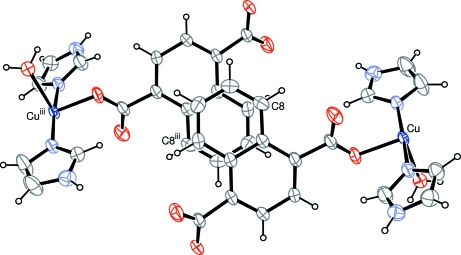
A digram showing the ovelapped arrangement of adjacent naphthaline ligands [symmetry code: (iii) 1 - x, 1 - y, 2 - z].

**Fig. 3.**
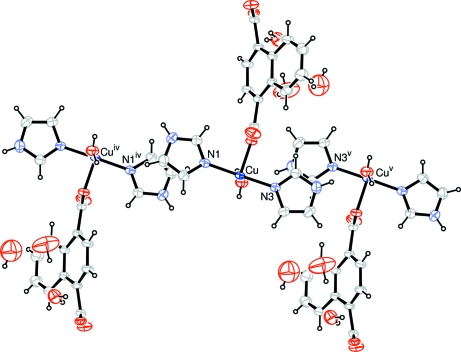
A diagram showing the contacts between imidazole rings [symmetry codes: (iv) 1 - x, -y, 1 - z; (v) 1 - x, 1 - y, 1 - z].

## supplementary crystallographic information

### Comment

As part of our investigation on the nature of π-π stacking between aromatic rings (Li *et al.*, 2005), the title polymeric complex of Cu^II^ incorporating imidazole and naphthalenedicarboxylate (naph) ligands has been prepared and its crystal structure is reported here.

The Cu^II^ cation is coordinated by two naph dianions, two imidazole molecules and one water molecule in a distorted square pyramidal geometry. The Cu—O(water) bond distance in the apical direction is longer than mean Cu—*O*(carboxyl) bond distance in the basal plane by 0.509 (3) Å. The naph dianion bridges two Cu^II^ cations by two carboxyl groups to form the one dimensional polymeric chain (Fig. 1). Two carboxyl groups of the naph dianion are twisted with respect to the C1-benzene ring with the dihedral angles of 32.0 (2)° and 38.2 (2)°, which are close to that found in the free naphthalenedicarboxylic acid (*ca* 40°; Derissen *et al.*, 1979) but are much smaller than those [52.5 (3)° and 48.7 (3)°] found in a Mn^II^ complex with the uncoordinated naph dianion (Li *et al.*, 2008). The coordinated water molecule (O5) is hydrogen bonded to carboxyl groups and imidazole ligand of adjacent polymeric chains (Table 2) to form the three dimensional supra-molecular structure.

The parallel C8-benzene and C8^iii^-benzene rings from the adjacent polymeric chains overlap as shown in Fig. 2 [symmetry code: (iii) 1 - *x*, 1 - *y*, 2 - *z*] with a face-to-face separation of 3.67 (2) Å  indicating no π-π stacking existing between benzene rings, a similar  situation to that found in the Mn^II^ complex with uncoordinated naph dianion (Li *et al.*, 2008). The face-to-face distances between parallel N1-imidazole and N1^iv^-imidazole rings and between parallel N3-imidazole and N3^v^-imidazole rings are 3.310 (4) and 3.050 (17) Å, respectively [symmetry codes: (iv) 1 - *x*, -*y*, 1 - *z*; (v) 1 - *x*, 1 - *y*, 1 - *z*]. However the imidazole rings are not overlapping each other in the crystal structure (Fig. 3), therefore no π-p\ stacking exists between parallel imidazole rings too.

### Experimental

A water-ethanol solution (12 ml, 1:1) containing naphthalene-1,4-dicarboxyllic acid (0.162 g, 0.75 mmol), sodium hydroxide (0.053 g, 1.3 mmol), sodium acetate trihydrate (0.204 g, 1.5 mmol), cupric chloride dihydrate (0.085 g, 0.5 mmol) and imidazole (0.034 g, 0.5 mmol) was refluxed for 3 h. After cooling to room temperature the solution was filtered. The single crystals of the title compound were obtained from the filtrate after 8 d.

### Refinement

The lattice water O2WB is close to an inversion center, while the lattice O2WA is *ca* 1.5 Å apart from O2WB^vi^ [symmetry code: (vi) -*x*, 1 - *y*, 1 - *z*]. The site occupancy factors of the O2WA and O2WB atoms were initially refined and converged to 0.48 and 0.45, and fixed as 0.50 for each at final cycles of refinemens. Water H atoms were placed in a difference Fourier map and refined in riding mode with *U*_iso_(H) = 1.5*U*_eq_(O). Other H atoms were placed in calculated positions with C—H = 0.93 Å and N—H = 0.86 Å, and refined in riding mode with *U*_iso_(H) = 1.2*U*_eq_(C,*N*).

### Crystal data

**Table:**

[Cu(C_12_H_6_O_4_)(C_3_H_4_N_2_)_2_(H_2_O)]·2H_2_O	*F*_000_ = 964
*M**_r_* = 467.92	*D*_x_ = 1.522 Mg m^−^^3^
Monoclinic, *P*2_1_/*n*	Mo *K*α radiation λ = 0.71073 Å
Hall symbol: -P 2yn	Cell parameters from 5466 reflections
*a* = 12.571 (2) Å	θ = 2.0–24.5º
*b* = 14.698 (3) Å	µ = 1.12 mm^−^^1^
*c* = 12.636 (2) Å	*T* = 295 (2) K
β = 119.011 (6)º	Prism, blue
*V* = 2041.8 (6) Å^3^	0.33 × 0.30 × 0.24 mm
*Z* = 4	

### Data collection

**Table:**

Rigaku R-AXIS RAPID IP diffractometer	3989 independent reflections
Radiation source: fine-focus sealed tube	3251 reflections with *I* > 2σ(*I*)
Monochromator: graphite	*R*_int_ = 0.043
Detector resolution: 10.0 pixels mm^-1^	θ_max_ = 26.0º
*T* = 295(2) K	θ_min_ = 1.9º
ω scans	*h* = −15→15
Absorption correction: multi-scan(ABSCOR; Higashi, 1995)	*k* = −18→17
*T*_min_ = 0.660, *T*_max_ = 0.765	*l* = −15→15
23205 measured reflections	

### Refinement

**Table:**

Refinement on *F*^2^	Secondary atom site location: difference Fourier map
Least-squares matrix: full	Hydrogen site location: inferred from neighbouring sites
*R*[*F*^2^ > 2σ(*F*^2^)] = 0.036	H-atom parameters constrained
*wR*(*F*^2^) = 0.097	*w* = 1/[σ^2^(*F*_o_^2^) + (0.0469*P*)^2^ + 1.2324*P*] where *P* = (*F*_o_^2^ + 2*F*_c_^2^)/3
*S* = 1.06	(Δ/σ)_max_ = 0.001
3989 reflections	Δρ_max_ = 0.50 e Å^−^^3^
280 parameters	Δρ_min_ = −0.39 e Å^−^^3^
Primary atom site location: structure-invariant direct methods	Extinction correction: none

### Special details

**Table:**

Geometry. All e.s.d.'s (except the e.s.d. in the dihedral angle between two l.s. planes) are estimated using the full covariance matrix. The cell e.s.d.'s are taken into account individually in the estimation of e.s.d.'s in distances, angles and torsion angles; correlations between e.s.d.'s in cell parameters are only used when they are defined by crystal symmetry. An approximate (isotropic) treatment of cell e.s.d.'s is used for estimating e.s.d.'s involving l.s. planes.
Refinement. Refinement of *F*^2^ against ALL reflections. The weighted *R*-factor *wR* and goodness of fit *S* are based on *F*^2^, conventional *R*-factors *R* are based on *F*, with *F* set to zero for negative *F*^2^. The threshold expression of *F*^2^ > σ(*F*^2^) is used only for calculating *R*-factors(gt) *etc*. and is not relevant to the choice of reflections for refinement. *R*-factors based on *F*^2^ are statistically about twice as large as those based on *F*, and *R*- factors based on ALL data will be even larger.

### Fractional atomic coordinates and isotropic or equivalent isotropic displacement parameters (Å^2^)

**Table:**

	*x*	*y*	*z*	*U*_iso_*/*U*_eq_	Occ. (<1)
Cu	0.61473 (3)	0.26395 (2)	0.57692 (3)	0.02692 (12)	
N1	0.60619 (19)	0.13328 (15)	0.6141 (2)	0.0324 (5)	
N2	0.5581 (2)	0.00805 (16)	0.6757 (2)	0.0408 (6)	
H2N	0.5252	−0.0276	0.7056	0.049*	
N3	0.64575 (19)	0.39456 (14)	0.56052 (19)	0.0310 (5)	
N4	0.6481 (2)	0.54324 (16)	0.5765 (2)	0.0423 (6)	
H4N	0.6401	0.5966	0.5997	0.051*	
O1	0.52250 (17)	0.29662 (13)	0.66172 (17)	0.0356 (4)	
O2	0.69612 (18)	0.29561 (17)	0.8353 (2)	0.0515 (6)	
O3	0.19639 (16)	0.27227 (12)	0.98022 (16)	0.0311 (4)	
O4	0.37130 (19)	0.25600 (14)	1.14909 (18)	0.0436 (5)	
O5	0.41477 (17)	0.27662 (13)	0.38636 (17)	0.0387 (5)	
H5A	0.3510	0.2580	0.3861	0.058*	
H5B	0.4082	0.2721	0.3166	0.058*	
O1W	0.0571 (2)	0.37164 (15)	0.7624 (2)	0.0548 (6)	
H1A	0.1072	0.3426	0.8274	0.082*	
H1B	0.0011	0.3328	0.7129	0.082*	
O2WA	0.1105 (11)	0.4187 (8)	0.5762 (10)	0.181 (5)	0.50
H2A	0.0710	0.4002	0.6169	0.271*	0.50
H2B	0.1794	0.3868	0.6144	0.271*	0.50
O2WB	−0.0010 (9)	0.5394 (6)	0.5243 (9)	0.127 (3)	0.50
H2C	0.0693	0.5188	0.5711	0.190*	0.50
H2D	−0.0381	0.5579	0.5650	0.190*	0.50
C1	0.5111 (2)	0.30793 (18)	0.8430 (2)	0.0294 (6)	
C2	0.3978 (2)	0.26894 (18)	0.7913 (2)	0.0325 (6)	
H2	0.3638	0.2466	0.7127	0.039*	
C3	0.3324 (2)	0.26212 (18)	0.8547 (2)	0.0327 (6)	
H3	0.2564	0.2343	0.8181	0.039*	
C4	0.3787 (2)	0.29591 (18)	0.9699 (2)	0.0283 (5)	
C5	0.5331 (3)	0.3954 (2)	1.1323 (2)	0.0376 (6)	
H5	0.4903	0.3923	1.1750	0.045*	
C6	0.6357 (3)	0.4476 (2)	1.1762 (3)	0.0441 (7)	
H6	0.6605	0.4810	1.2468	0.053*	
C7	0.7035 (3)	0.4508 (2)	1.1152 (3)	0.0452 (7)	
H7	0.7740	0.4856	1.1463	0.054*	
C8	0.6671 (3)	0.4035 (2)	1.0115 (3)	0.0399 (7)	
H8	0.7144	0.4053	0.9733	0.048*	
C9	0.5581 (2)	0.35118 (17)	0.9593 (2)	0.0287 (5)	
C10	0.4903 (2)	0.34582 (17)	1.0226 (2)	0.0284 (5)	
C11	0.5842 (3)	0.30043 (18)	0.7771 (3)	0.0329 (6)	
C12	0.3125 (2)	0.27394 (17)	1.0399 (2)	0.0289 (6)	
C13	0.6235 (2)	0.46473 (19)	0.6125 (2)	0.0352 (6)	
H13	0.5945	0.4598	0.6674	0.042*	
C14	0.6877 (3)	0.5244 (2)	0.4974 (3)	0.0585 (9)	
H14	0.7112	0.5661	0.4572	0.070*	
C15	0.6865 (3)	0.4329 (2)	0.4878 (3)	0.0561 (9)	
H15	0.7099	0.4006	0.4391	0.067*	
C16	0.5431 (3)	0.0979 (2)	0.6621 (3)	0.0418 (7)	
H16	0.4940	0.1315	0.6838	0.050*	
C17	0.6345 (3)	−0.0168 (2)	0.6339 (3)	0.0405 (7)	
H17	0.6612	−0.0754	0.6316	0.049*	
C18	0.6642 (3)	0.05965 (19)	0.5964 (3)	0.0389 (6)	
H18	0.7161	0.0627	0.5635	0.047*	

### Atomic displacement parameters (Å^2^)

**Table:**

	*U*^11^	*U*^22^	*U*^33^	*U*^12^	*U*^13^	*U*^23^
Cu	0.03055 (19)	0.02963 (19)	0.03180 (19)	0.00027 (12)	0.02397 (15)	−0.00133 (13)
N1	0.0350 (12)	0.0336 (13)	0.0378 (12)	0.0004 (10)	0.0250 (10)	−0.0019 (10)
N2	0.0484 (14)	0.0342 (14)	0.0467 (14)	−0.0069 (11)	0.0287 (12)	0.0010 (11)
N3	0.0341 (12)	0.0315 (12)	0.0365 (12)	0.0011 (9)	0.0241 (10)	0.0009 (9)
N4	0.0448 (14)	0.0314 (13)	0.0511 (15)	−0.0005 (10)	0.0234 (12)	−0.0021 (11)
O1	0.0446 (11)	0.0381 (11)	0.0412 (11)	0.0005 (8)	0.0344 (10)	−0.0015 (8)
O2	0.0333 (12)	0.0736 (15)	0.0599 (14)	0.0010 (10)	0.0323 (11)	−0.0102 (11)
O3	0.0295 (10)	0.0387 (10)	0.0359 (10)	−0.0006 (8)	0.0243 (9)	0.0022 (8)
O4	0.0398 (11)	0.0662 (14)	0.0322 (11)	0.0016 (10)	0.0233 (9)	0.0083 (9)
O5	0.0379 (11)	0.0476 (12)	0.0360 (10)	−0.0086 (9)	0.0222 (9)	−0.0074 (9)
O1W	0.0509 (13)	0.0544 (14)	0.0585 (14)	0.0047 (11)	0.0261 (11)	−0.0028 (11)
O2WA	0.216 (12)	0.203 (12)	0.214 (12)	−0.016 (9)	0.175 (11)	0.029 (9)
O2WB	0.117 (6)	0.131 (8)	0.126 (7)	−0.021 (6)	0.054 (6)	−0.012 (6)
C1	0.0315 (14)	0.0311 (14)	0.0351 (14)	0.0024 (11)	0.0236 (12)	0.0008 (11)
C2	0.0341 (15)	0.0403 (16)	0.0315 (14)	−0.0037 (11)	0.0225 (12)	−0.0070 (11)
C3	0.0287 (14)	0.0423 (16)	0.0350 (14)	−0.0053 (11)	0.0218 (12)	−0.0046 (12)
C4	0.0290 (13)	0.0336 (14)	0.0311 (13)	0.0035 (10)	0.0215 (11)	0.0028 (11)
C5	0.0421 (16)	0.0464 (17)	0.0325 (14)	−0.0013 (13)	0.0245 (13)	−0.0036 (12)
C6	0.0501 (18)	0.0453 (18)	0.0374 (16)	−0.0077 (14)	0.0217 (14)	−0.0098 (13)
C7	0.0369 (16)	0.0500 (19)	0.0490 (18)	−0.0146 (13)	0.0211 (14)	−0.0088 (14)
C8	0.0360 (15)	0.0437 (17)	0.0478 (17)	−0.0077 (13)	0.0265 (14)	−0.0009 (13)
C9	0.0290 (13)	0.0304 (14)	0.0329 (13)	0.0021 (10)	0.0200 (11)	0.0016 (10)
C10	0.0309 (13)	0.0295 (14)	0.0304 (13)	0.0027 (10)	0.0192 (11)	0.0016 (10)
C11	0.0417 (17)	0.0273 (14)	0.0469 (17)	−0.0020 (11)	0.0351 (14)	−0.0013 (11)
C12	0.0332 (14)	0.0292 (14)	0.0345 (14)	0.0018 (11)	0.0245 (12)	−0.0001 (11)
C13	0.0354 (15)	0.0381 (16)	0.0348 (14)	−0.0012 (12)	0.0192 (12)	−0.0017 (12)
C14	0.080 (2)	0.0393 (19)	0.087 (3)	0.0010 (16)	0.065 (2)	0.0105 (17)
C15	0.087 (3)	0.0380 (18)	0.081 (2)	0.0069 (16)	0.070 (2)	0.0094 (16)
C16	0.0528 (18)	0.0339 (16)	0.0551 (18)	−0.0020 (13)	0.0390 (16)	−0.0023 (13)
C17	0.0414 (16)	0.0339 (16)	0.0454 (16)	0.0028 (12)	0.0206 (14)	0.0011 (13)
C18	0.0403 (16)	0.0370 (16)	0.0467 (17)	0.0057 (12)	0.0268 (14)	0.0023 (13)

### Geometric parameters (Å, °)

**Table:**

Cu—N1	1.992 (2)	C1—C2	1.371 (4)
Cu—N3	1.990 (2)	C1—C9	1.439 (4)
Cu—O1	1.9819 (17)	C1—C11	1.515 (3)
Cu—O3^i^	2.0116 (17)	C2—C3	1.404 (4)
Cu—O5	2.506 (2)	C2—H2	0.9300
N1—C16	1.317 (3)	C3—C4	1.372 (4)
N1—C18	1.382 (3)	C3—H3	0.9300
N2—C16	1.333 (4)	C4—C10	1.430 (4)
N2—C17	1.351 (4)	C4—C12	1.514 (3)
N2—H2N	0.8600	C5—C6	1.365 (4)
N3—C13	1.323 (3)	C5—C10	1.421 (4)
N3—C15	1.372 (4)	C5—H5	0.9300
N4—C13	1.331 (4)	C6—C7	1.400 (4)
N4—C14	1.344 (4)	C6—H6	0.9300
N4—H4N	0.8600	C7—C8	1.352 (4)
O1—C11	1.278 (3)	C7—H7	0.9300
O2—C11	1.234 (3)	C8—C9	1.424 (4)
O3—C12	1.277 (3)	C8—H8	0.9300
O3—Cu^ii^	2.0116 (17)	C9—C10	1.426 (3)
O4—C12	1.237 (3)	C13—H13	0.9300
O5—H5A	0.8450	C14—C15	1.351 (5)
O5—H5B	0.8478	C14—H14	0.9300
O1W—H1A	0.8670	C15—H15	0.9300
O1W—H1B	0.8864	C16—H16	0.9300
O2WA—H2A	0.9128	C17—C18	1.340 (4)
O2WA—H2B	0.8933	C17—H17	0.9300
O2WB—H2C	0.8461	C18—H18	0.9300
O2WB—H2D	0.8876		
			
O1—Cu—N3	91.04 (8)	C6—C5—H5	119.3
O1—Cu—N1	89.64 (8)	C10—C5—H5	119.3
N3—Cu—N1	172.02 (9)	C5—C6—C7	120.2 (3)
O1—Cu—O3^i^	175.67 (8)	C5—C6—H6	119.9
N3—Cu—O3^i^	90.49 (8)	C7—C6—H6	119.9
N1—Cu—O3^i^	89.41 (8)	C8—C7—C6	120.4 (3)
O5—Cu—N1	98.89 (8)	C8—C7—H7	119.8
O5—Cu—N3	89.09 (8)	C6—C7—H7	119.8
O5—Cu—O1	85.66 (7)	C7—C8—C9	121.6 (3)
O5—Cu—O3^i^	90.32 (7)	C7—C8—H8	119.2
C16—N1—C18	104.3 (2)	C9—C8—H8	119.2
C16—N1—Cu	127.07 (19)	C8—C9—C10	118.2 (2)
C18—N1—Cu	128.61 (18)	C8—C9—C1	122.6 (2)
C16—N2—C17	107.4 (2)	C10—C9—C1	119.1 (2)
C16—N2—H2N	126.3	C5—C10—C9	118.2 (2)
C17—N2—H2N	126.3	C5—C10—C4	122.6 (2)
C13—N3—C15	104.5 (2)	C9—C10—C4	119.1 (2)
C13—N3—Cu	126.94 (18)	O2—C11—O1	124.2 (2)
C15—N3—Cu	128.44 (19)	O2—C11—C1	119.8 (2)
C13—N4—C14	107.9 (3)	O1—C11—C1	115.9 (2)
C13—N4—H4N	126.0	O4—C12—O3	123.3 (2)
C14—N4—H4N	126.0	O4—C12—C4	119.7 (2)
C11—O1—Cu	115.93 (16)	O3—C12—C4	117.0 (2)
C12—O3—Cu^ii^	114.83 (16)	N3—C13—N4	111.5 (2)
H5A—O5—H5B	110.9	N3—C13—H13	124.3
H1A—O1W—H1B	108.4	N4—C13—H13	124.3
H2A—O2WA—H2B	100.9	N4—C14—C15	106.2 (3)
H2C—O2WB—H2D	111.6	N4—C14—H14	126.9
C2—C1—C9	119.3 (2)	C15—C14—H14	126.9
C2—C1—C11	118.3 (2)	C14—C15—N3	109.9 (3)
C9—C1—C11	122.4 (2)	C14—C15—H15	125.0
C1—C2—C3	121.2 (2)	N3—C15—H15	125.0
C1—C2—H2	119.4	N1—C16—N2	111.8 (3)
C3—C2—H2	119.4	N1—C16—H16	124.1
C4—C3—C2	121.0 (2)	N2—C16—H16	124.1
C4—C3—H3	119.5	C18—C17—N2	106.5 (3)
C2—C3—H3	119.5	C18—C17—H17	126.7
C3—C4—C10	119.7 (2)	N2—C17—H17	126.7
C3—C4—C12	118.1 (2)	C17—C18—N1	109.9 (2)
C10—C4—C12	122.1 (2)	C17—C18—H18	125.1
C6—C5—C10	121.4 (2)	N1—C18—H18	125.1

Symmetry codes: (i) *x*+1/2, −*y*+1/2, *z*−1/2; (ii) *x*−1/2, −*y*+1/2, *z*+1/2.

### Hydrogen-bond geometry (Å, °)

**Table:**

*D*—H···*A*	*D*—H	H···*A*	*D*···*A*	*D*—H···*A*
O1W—H1A···O3	0.87	1.99	2.846 (3)	170
O1W—H1B···O4^iii^	0.89	1.93	2.789 (3)	162
O2WA—H2A···O1W	0.91	1.97	2.828 (13)	155
O2WB—H2C···O2WA	0.85	1.55	2.156 (16)	126
N2—H2N···O1W^iv^	0.86	1.96	2.798 (4)	165
N4—H4N···O5^v^	0.86	2.02	2.866 (3)	166
O5—H5A···O2^iii^	0.85	1.90	2.716 (3)	162
O5—H5B···O4^vi^	0.85	1.95	2.791 (3)	172
C17—H17···O2^vii^	0.93	2.50	3.389 (4)	160

Symmetry codes: (iii) *x*−1/2, −*y*+1/2, *z*−1/2; (iv) −*x*+1/2, *y*−1/2, −*z*+3/2; (v) −*x*+1, −*y*+1, −*z*+1; (vi) *x*, *y*, *z*−1; (vii) −*x*+3/2, *y*−1/2, −*z*+3/2.
